# Isolation and molecular characterization of type I and type II feline coronavirus in Malaysia

**DOI:** 10.1186/1743-422X-9-278

**Published:** 2012-11-21

**Authors:** Alazawy Amer, Arshad Siti Suri, Omar Abdul Rahman, Hair Bejo Mohd, Bande Faruku, Sharif Saeed, Tengku Ibrahim Tengku Azmi

**Affiliations:** 1 Department of Veterinary Pathology and Microbiology, Faculty of Veterinary Medicine, Universiti Putra Malaysia, UPM Serdang, Selangor 43400, Malaysia; 2 Iraq, Ministry of Higher Education & Scientific Research, College of Veterinary Medicine, University of Diyala, Diyala, Iraq; 3 Department of Veterinary Preclinical Sciences, Faculty of Veterinary Medicine, Universiti Putra Malaysia, UPM Serdang, Selangor, 43400, Malaysia

**Keywords:** FCoV, FIPV, CrFK, Fcwf-4, S-gene, Phylogenetics, Cats, Malaysia

## Abstract

**Background:**

Feline infectious peritonitis virus (FIPV) and feline enteric coronavirus (FECV) are two important coronaviruses of domestic cat worldwide. Although FCoV is prevalent among cats; the fastidious nature of type I FCoV to grow on cell culture has limited further studies on tissue tropism and pathogenesis of FCoV. While several studies reported serological evidence for FCoV in Malaysia, neither the circulating FCoV isolated nor its biotypes determined. This study for the first time, describes the isolation and biotypes determination of type I and type II FCoV from naturally infected cats in Malaysia.

**Findings:**

Of the total number of cats sampled, 95% (40/42) were RT-PCR positive for FCoV. Inoculation of clinical samples into Crandell feline kidney cells (CrFK), and Feline *catus whole fetus-4* cells (Fcwf-4), show cytopathic effect (CPE) characterized by syncytial cells formation and later cell detachment. Differentiation of FCoV biotypes using RT-PCR assay revealed that, 97.5% and 2.5% of local isolates were type I and type II FCoV, respectively. These isolates had high sequence homology and phylogenetic similarity with several FCoV isolates from Europe, South East Asia and USA.

**Conclusions:**

This study reported the successful isolation of local type I and type II FCoV evident with formation of cytopathic effects in two types of cell cultures namely the CrFK and Fcwf-4 , where the later cells being more permissive. However, the RT-PCR assay is more sensitive in detecting the antigen in suspected samples as compared to virus isolation in cell culture. The present study indicated that type I FCoV is more prevalent among cats in Malaysia.

## Findings

Feline coronavirus (FCoV) is an enveloped RNA virus belonging to *Coronaviridae.* The disease is prevalent especially in catteries and multiple-cat household [1]*.* FCoV consist of two biotypes; Feline infectious peritonitis (FIPV) which is highly fatal and caused immune mediated complex, and feline enteric coronavirus (FECV) which caused from asymptomatic infection to severe enteritis [1,2].

Two types of FCoV could be distinguished based on serology and sequence analysis. Type I FCoV is wholly feline-associated and prevalent in natural cases of coronavirus infections but it poorly proliferates in cell culture. On the other hand, type II FCoV is less common but grows in different cell lines such as Fcwf-4 and CrFK cells [3,4].

Although evidence of FIP have been reported among cat population in Malaysia [5,6], the circulating FCoV virus neither isolated nor characterized. Thus, the objectives of this study are to isolate and determine the FCoV biotypes occurring in Malaysia.

Of the 42 RNA samples used in this study, 40 samples (95%) were positive and 2 samples (5%) were negative for FCoV following RT-PCR assay. Of these, RT-PCR positive samples, 95% (40/42) and 73.8% (31/42) were able to adapt and multiply in Fcwf-4 and CrFK cell cultures, respectively. Two samples which were positive with RT-PCR assay were found not to grow in cell culture. The entire 40 tissue culture adapted virus were used in the subsequent studies.

**Figure 1:**
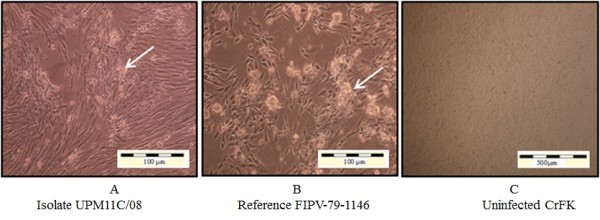
**CrFK-4 cell cultures showing cytopathic changes following infection with reference and local FCoV isolate UPM 11C/08. **Morphology of cells infected with local FCoV UPM 11C/08 isolate at passage 3 (**A**). where the initial CPE was observed at passage 2. At fifth viral passages, complete CPE occurred within 48-72hours PI. Morphology of cells infected with reference FIPV-79-1146 strain (**B**). Infected cells show CPE characterized by cells rounding, clumped and detachment (arrows). Note that the changes in cells infected with UPM11C/08 are comparable to reference strain. Control uninfected cells remained normal (**C**). Unstained.

**Figure 2:**
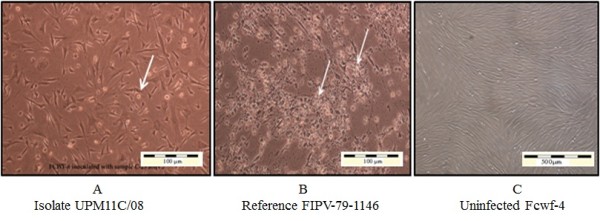
**Fcwf-4 cell cultures showing cytopathic changes following infection with reference and local FCoV isolate UPM 11C/08. **Morphology of cells infected with local FCoV UPM 11C/08 isolate at passage 3 (**A**). where the initial CPE was observed at passage 1. At third viral passages, complete CPE occurred within 24 hours PI. Morphology of cells infected with reference FIPV-79-1146 strain. (**B**). The infected cells show CPE characterized by cells rounding, clumped and detachment (arrows). Note that the changes in cells infected with UPM11C/08 are comparable to reference strain. Control uninfected cells remained normal (**C**). Unstained.

Upon virus inoculation, infected CrFK cells showed initial CPE at passage two with moderate to diffuse CPE at 4–5 days post inoculation (PI). The appearance of CPE became rapid during second and third passages. As the virus propagation reaches the fifth passages; the onset of CPE appeared within 24–48 hours PI. Complete CPE (> 80%) was noticed 48–72 hours PI (Figure 1). On the other hand, infected Fcwf-4 cells showed initial CPE following first passage, with some samples at second passages. The initial CPE was recorded at 2–3 days PI. As the virus passages reach the third passages, the onset of CPE appeared within 24 hours PI and completed within 36–48 hours PI (Figure 2).

**Figure 3:**
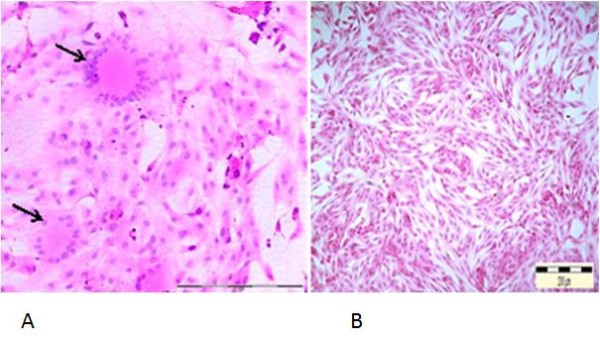
**Syncytial cells formation following infection of Fcwf-4 cell culture with FCoV UPM 11C/08 isolate. **Infected cells show loss of plasma membrane and nuclear aggregation, resulting in formation of syncytial cells containing more than 20 nuclei (arrows), 36 hours PI. 20x Mag. Scale bar, 100 μ m. Normal uninfected Fcwf-4 cell culture at 72 hours. H&E staining (**A**). Cells appeared as spindle to stellate morphology and regularly arranged. 10x Mag. Scale bar, 200 μm (**B**).

Both infected CrFK and Fcwf-4 cells showed similar morphological changes characterized by increased opacity and refractile of the infected cells. Infected cells become rounded, granular and clumped forming syncytial cells which increased in size and number as the incubation period extended. Syncytial cells were observed to contain between 20–30 nuclei per cell at 36–48 hours PI, although 8–10 nuclei per cell was most common (Figure 3). Morphological changes observed in CrFK and Fcwf-4 following infection with FCoV isolates were comparable to reference FIPV-79-1146. Both control uninfected cells remained normal.

**Figure 4:**
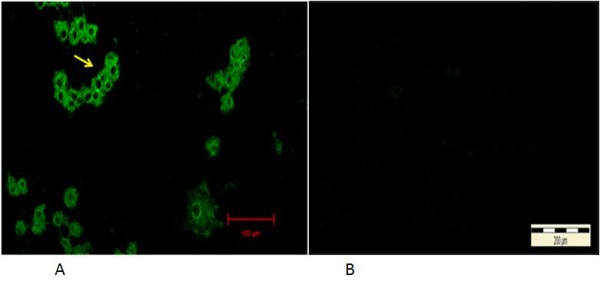
**Immunofluorescent antibodies staining of infected CrFK cells following infection with local FCoV UPM11C/08 isolate. **The fluorescence signal appeared as granules covering areas within the cytoplasm at 24 hours PI (arrow). No signal is present in the nucleus. 100x Mag. Scale bar, 20μm (**A**). Absence of fluorescence signal in control uninfected cell cultures. 10x Mag. Bar = 200μm (**B**).

Indirect immunofluorescent antibody test (IIF) on infected cells showed viral antigens were present in the cytoplasm, where the earliest signals appeared as fine to coarse immunofluorescent granules in the perinuclear region of the infected cells at 6 hours PI (Figure 4).

Differentiation of FCoV using specific primer sets for type I (fecv1b) and type II FCoV (fecv2b) yielded an expected amplification product of 275 bp and 232 bp denoting to type I FCoV (n = 39/40) and type II FCoV (n= 1/40), respectively ( data not shown). A total of 13 selected RT-PCR products samples, each from type I (n_1_=12) and type II (n_2_=1) local FCoV were sequenced for compared with reference strains and subjected for phylogenetic analysis.

**Figure 5:**
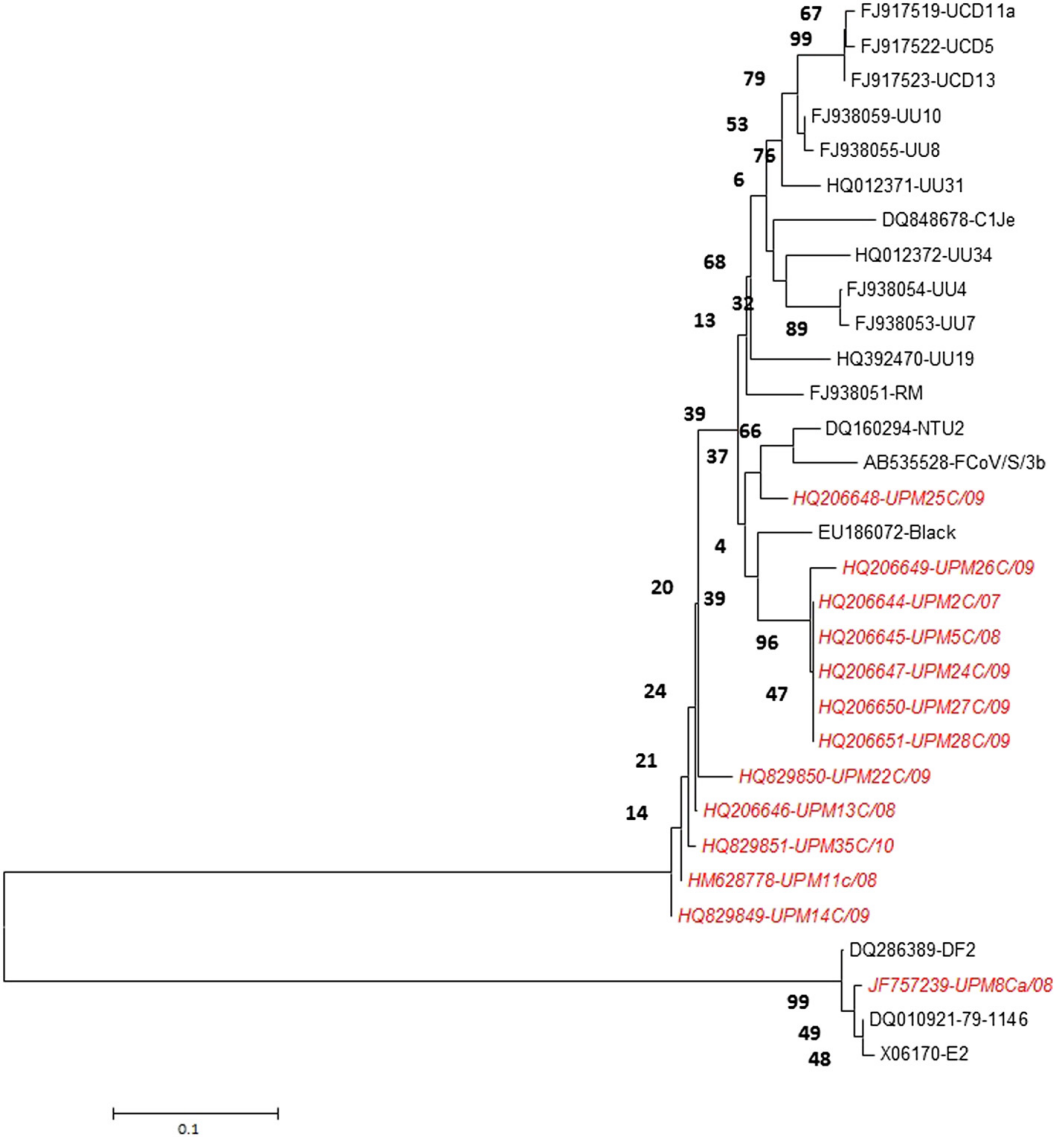
**Phylogenetic tree depicting relationships between Malaysian FCoV and reference FCoV isolates based on partial S gene sequence. **The tree was generated using MEGA5 program. The tree reliability was calculated using 1000 bootstrapped replicates. Malaysian FCoVs are indicated in red italic.

The findings from phylogenetic analysis of local FCoV further support their biotyping where majority of local FCoVs (n=12) closely branched together with type I reference FCoV and only one sample; UPM8Ca/08 was found to cluster with type II FCoV (Figure 5). The nucleotide sequences and accession numbers of isolates derived from this study have been deposited in the NCBI GenBank database (Table 1).

**Table 1:** List of sequences used in the phylogenetic analysis of Malaysian FCOVs isolates

**Isolate/ Strain**	**Accession No.**	**Country**	**Nucleotide position used**
UPM2C/07	HQ206644	Malaysia	1-224
UPM5C/08	HQ206645	Malaysia	1-224
UPM11C/08	HM628778	Malaysia	1-221
UPM13C/08	HQ206646	Malaysia	1-224
UPM8Ca/08	JF757239	Malaysia	1-199
UPM14C/09	HQ829849	Malaysia	1-224
UPM22C/09	HQ829850	Malaysia	1-224
UPM24C/09	HQ206647	Malaysia	1-224
UPM25C/09	HQ206648	Malaysia	1-224
UPM26C/09	HQ206649	Malaysia	1-221
UPM27C/09	HQ206650	Malaysia	1-224
UPM28C/09	HQ206651	Malaysia	1-224
UPM35C/10	HQ829851	Malaysia	1-224
FCoV Black	EU186072	USA	24623-24846
FCoV UU19	HQ392470	Netherlands	24610-24830
FCoV RM	FJ938051	USA	24572-24792
FCoV UU31	HQ012371	Netherlands	24613-24833
FCoV UU10	FJ938059	Netherlands	24626-24849
FIPV UCD11a	FJ917519	USA	4290-4513
FCoV UU8	FJ938055	Netherlands	24613-24836
FCoV UU4	FJ938054	Netherlands	24620-24843
FCoV UU7	FJ938053	Netherlands	24620-24843
FCoV/NTU2	DQ160294	Taiwan	4294-4517
FCoV UU34	HQ012372	Netherlands	24562-24785
FCoV/S/3b	AB535528	Japan	3568-3791
FIPV UCD13	FJ917523	USA	4316-4539
FCoV C1Je	DQ848678	UK	24616-24839
FECV UCD5	FJ917522	USA	4316-4539
FIPV 79-1146	DQ010921	USA	24507-24691
FIPV DF2	DQ286389	USA	24737-24921
FIPVE2	X06170	Italy	4371- 4500

Comparison amongst local FCoV type I sequences revealed high degree of sequence homology, ranging from 93.2-99.5%. However, this similarity decreases to 90% when compared with the reference isolates. In contrast, type II FCoV isolates (UPM8Ca/08) only shows sequence identity ranging from 77.6-79% when compared with reference isolates. Malaysian FCoV type I isolates were phylogenetically closer to FCoV isolates from America, South East Asia, and Europe while UPM8Ca/08 which is the only identified type II isolates, was closer to FIPV 79–1146 and FIPV DF2 reference isolates originating from USA (Figure 5).

In this study, both RT-PCR and virus isolation were employed to detect the presence of FCoV from naturally infected domestic cats. The FCoV detection rate (95%) observed in this study is comparably higher than reported by Herrewegh et al. [7].

Based on partial S-gene sequence, the circulating virus in Malaysia was found to be predominately of type I FCoV (39/40). High incidence of type I FCoV (97.5%) in comparison to type II FCoV (2.5%) is in agreement with other findings reported in countries like Japan, Australia and Korea [8-10]. Similarly, the sequence homology observed among local FCoV isolates were in agreement with Lin et al*.*[11].

Comparing for virus adaptation in two cell lines revealed that Fcwf-4 was more permissive to local FCoV replication. Thus, all local FCoV type I isolates (39/39) were able to adapt and grow in Fcwf-4 cells as compared to only 77% (30/39) samples that grow on CrFK cells (data not shown). This finding further support the predilection of type I FCoV towards macrophages [4,12]. Replication of the virus is restricted in the cytoplasm of the infected cells as indicated by the presence of the fluorescent signals with the production of virus particle typical of coronavirus (13).

In conclusion, this study described the isolation of FCoV in feline cell culture from domestic cats in Malaysia. The isolates replicate in CrFK and Fcwf-4 cell cultures with the formation of CPE comparable to reference strain. Phylogenetic analyses show that majority of the local FCoV isolates belongs to type I and rarely of type II biotypes. Additionally, the study revealed that, Malaysia FCoV s is phylogenetically related with isolates from South East Asia, USA and UK, signifying possible common ancestral origin.

## Material and methods

Ascitic fluid sample (n= 27) and postmortem tissues (n=15) were collected from 42 clinically ill client-owned presented at University Veterinary Hospital, Universiti Putra Malaysia (UVH -UPM) during 4-year period (2007–2010). The sampled cats comprises of 28 male and 14 females with age range from 6 month to 5 years. All cats came from neibouring areas within Klang valley and had no previous history of vaccination against FCoV as FIP vaccination is not a common practice in most clinics. Samples were consecutively designated as FCoV UPM1C/07 to UPM42C/10. The samples were processed and inoculated onto the CrFK (ATCC^®^; CCL-94) and Fcwf-4 (ATCC; CRL-2787) as described previously [13]. Uninfected CrFK and Fcwf-4 cells were used as negative control. The study was approved by Institution’s Animal Care and Use Committee, UPM (AUP No. 08R47).

Haematoxylin and eosin (H&E) staining of cell cultures was carried as described by Hsiung [14]. On the other hand, indirect immunofluorescent (IIF) test using polyclonal antibodies against FIPV 79–1146 virus was performed according to the method of Evermann et al. [15].

Viral RNA was extracted from infected cell cultures or Ascites fluid using Trizol Reagent (Invitrogen, USA) according to manufacturer’s instructions. RT-PCR was performed using Access RT-PCR system (Promega, USA) [6] with previously described primers p205 and p211 [16]. Positive samples were further differentiated into type I and type II FCoV using biotypes specific oligonucleotide primers targeting S-gene region [17]. All primers were synthesized by First Base Laboratories Sdn Bhd, Selangor, Malaysia.

To gain insight on the genetic relatedness of Malaysian FCoV isolates, the partial S-gene sequences of randomly selected RT-PCR positive samples (n=13) were sequenced using the ABI BigDye^®^ Terminator v3.1 cycle sequencing ready reaction kit (Applied Bioscience). Nucleotide sequences were compared with the corresponding reference FCoV isolates using BLAST software available in NCBI database. Phylogenetic tree was constructed with MEGA5 software using Neighbor Joining tree method. The reliability of the phylogenetic trees was calculated using 1000 bootstrap [18] (Figure 5).

## Abbreviations

FCoV: Feline coronavirus virus; RT-PCR: Reverse transcriptase polymerase chain reaction; RNA: Ribonucleic acid; LTR: Long terminal repeat; NCBI: National Centre for Biotechnology Information; UPM: Universiti Putra Malaysia.

## Competing interests

The authors declare that they have no competing interest.

## Authors’ contributions

AA: carried out the study and prepared the manuscript; SSA: Conceived the study, edited the manuscript and approved final copy for submission; OAR and MHB, participated in the study design and proof-read the manuscript; FB: carried out phylogenetic analysis, participated in manuscript drafting, proof-reading and submissions. SS: Optimized RT-PCR method for FCoV screening. All authors have read and approved final manuscript.

## References

[B1] Addie DD Jarrett JO A study of naturally occurring feline coronavirus infection in kittens Vet Rec 1992 130 133 137 10.1136/vr.130.7.133 1313617

[B2] Boyle JF Pedersen NC Evermann JF McKeirnan AJ Ott RL Black JW Plaque assay, polypeptide composition and immunochemistry of feline infectious peritonitis virus and feline enteric coronavirus isolates Adv Exp Med Biol 1984 173 133 147 10.1007/978-1-4615-9373-7_12 6331106

[B3] Motokawa K Hohdatsu T Aizawa C Koyama H Hashimoto H Molecular cloning and sequence determination of the peplomer protein gene of feline infectious peritonitis virus type I Arch Virol 1995 140 469 480 10.1007/BF01718424 7733820 PMC7086962

[B4] Pedersen NC A review of feline infectious peritonitis virus infection: 1963–2008 J Feline Med Surg 2009 11 225 258 10.1016/j.jfms.2008.09.008 19254859 PMC7129802

[B5] Arshad SS Lee WW Hassan L Kamarudin AIM Siti-Farawahida AW Cheng NABY Serological survey of catteries for cats infected with feline coronavirus J Vet Malaysia 2004 17 19 22

[B6] Sharif S Arshad SS Hair-Bejo M Omar AR Zeenathul NA Fong LS Descriptive distribution and phylogenetic analysis of feline infectious peritonitis virus isolates of Malaysia Acta Vet Scandinavica 2010 52 1 7 10.1186/1751-0147-52-1 PMC2828449 20053278

[B7] Herrewegh AAPM Smeenk I Horzinek MC Rottier PJM de Groot RJ Feline coronavirus type II strains 79–1683 and 79–1146 originate from a double recombination between feline coronavirus type I and canine coronavirus J Virol 1998 72 4508 4520 9557750 10.1128/jvi.72.5.4508-4514.1998 PMC109693

[B8] Hohdatsu T Okada S Ishizuka Y Yamada H Koyama H The prevalence of types I and II feline coronavirus infections in cats J Vet Med Sci 1992 54 557 562 10.1292/jvms.54.557 1322718

[B9] Benetka V Kubber-Heiss A Kolodziejek J Nowotny N Hofmann-Parisot M Mostl K Prevalence of feline coronavirus types I and II in cats with histopathologically verified feline infectious peritonitis Vet Microbio 2004 99 31 42 10.1016/j.vetmic.2003.07.010 PMC7117137 15019109

[B10] An DJ Jeoung HY Jeong W Park JY Lee MH Park BK Prevalence of Korean cats with natural feline coronavirus infections Virol J 2011 8 455 10.1186/1743-422X-8-455 21951835 PMC3219666

[B11] Lin CN Su BL Wang CH Hsieh MW Chueh TJ Chueh LL Genetic diversity and correlation with feline infectious peritonitis of feline coronavirus type I and II: a 5-year study in Taiwan Vet Microbiol 2009 136 233 239 10.1016/j.vetmic.2008.11.010 19117699 PMC7117496

[B12] Pedersen NC An overview of feline enteric coronavirus and infectious peritonitis virus infections Feline Pract 1995 23 7 20

[B13] Alazawy A Arshad SS Bejo MH Omar AR Tengku Ibrahim TA Sharif S Bande F Awang-Isa K Ultrastructure of Felis catus whole fetus (Fcwf-4) cell culture following infection with feline coronavirus J Electron Microsc 2011 60 275 282 10.1093/jmicro/dfr031 PMC7793022 21593079

[B14] Hsiung GD Diagnostic virology- an illustrated handbook 1973 New Haven and London: Yale University Press

[B15] Evermann JF Heeney JL McKeirnan AJ O'Brien SJ Comparative features of a coronavirus isolated from a cheetah with feline infectious peritonitis Virus Res 1989 13 15 27 10.1016/0168-1702(89)90084-1 2546331 PMC7133882

[B16] Herrewegh AAPM De Groot R Cepica A Egberink HF Horzinek MC Rottier P Detection of feline coronavirus RNA in feces, tissues, and body fluids of naturally infected cats by reverse transcriptase PCR J Clin Microbio 1995 33 684 695 10.1128/jcm.33.3.684-689.1995 PMC228014 7751377

[B17] Posch A Posch U Kubber-Heiss A Seiser M Moestl K Differentiation of feline coronaviruses type I and II strains by RT-PCR Proceedings of the WSAVA World veterinary congress 1999 Lyon

[B18] Felsenstein J Confidence limits on phylogenies: an approach using the bootstrap Evolution 1985 39 783 791 10.2307/2408678 28561359

